# Proteomic Analysis of Larval Midgut from the Silkworm (*Bombyx mori*)

**DOI:** 10.1155/2011/876064

**Published:** 2011-05-18

**Authors:** Sai Zhang, Yunmin Xu, Qiang Fu, Ling Jia, Zhonghuai Xiang, Ningjia He

**Affiliations:** The Key Sericultural Laboratory of Agricultural Ministry, College of Biotechnology, Southwest University, Beibei, Chongqing 400715, China

## Abstract

The midgut is the major organ for food digestion, nutrient absorption and also a barrier for foreign substance. The 5th-instar larval stage of silkworm is very important for larval growth, development, and silk production. In the present study, we used 2-DE and matrix-assisted laser desorption/ionization time-of-flight mass spectrometry (MALDI-TOF-MS) to analyze the midgut proteins from the 5th-instar larvae as well as the midgut proteins under starvation condition. A total of 96 proteins were identified in this study; and among them, 69 proteins were observed in midgut for the first time. We also found that the silkworm larval midgut responded to starvation by producing a 10 kDa heat shock protein and a diapause hormone precursor.

## 1. Introduction

Silkworm, *Bombyx mori*, is a domesticated insect for silk production. It is a lab-reared animal, and its relatively large size allows silkworm to be an important model in molecular genetics and in structural and functional genomics [1]. In addition, the silkworm also is a phytophagous insect and therefore a representative of lepidopteran pest insects. 

The silkworm, as a holometabolous insect, has four remarkably different developmental stages: egg, larva, pupa, and moth. The larva is the only stage in which the animals feed and excrete in the whole life cycle. This stage, especially the 5th instar, is very important because the larvae have enough nutrient for growth, development, and silk production. The larval midgut is formed of an epithelial cell monolayer composed of columnar, goblet, and stem cells [2]. The columnar cells are mainly responsible for food digestion and nutrition absorption. Digestion is usually controlled by digestive enzymes and is dependent on their localization in gut [3]. The apical membrane of columnar cells is characterized by a well-developed brush border [4]. Goblet cells participate in the ionic regulation in gut, and the regenerative cells are responsible for renewing epithelial cells during metamorphosis [5].

Midgut is also a barrier to the foreign substances during food digestion. It has been found that some proteins such as lipase [6] and SP-2 [7] in midgut have antiviral activity against *Bombyx mori* nuclear polyhedrosis virus (BmNPV). Moreover, midgut has early been recognized as one of the important targets for insect control. One successful example is the transgenic crops that produce *B. thuringiensis* crystal *δ*-endotoxins. These toxins bind to their receptors and then form a prepore oligomeric structure through which cell content leaking leads to the death of insect [8]. An alternative method for more specific control of insect is to silence the expression of genes using RNA interference (RNAi) through the midgut [9]. 

In order to understand the molecular mechanisms for nutrition digestion and midgut-derived defense, Yao et al. [10] and Kajiwara et al. [11] separately reported the proteomic analysis of the silkworm larval midgut. The former analysis was only performed on the 5th-instar day-3 silkworm larvae, and the later work focused on the midgut proteins from 5th-instar day-2 larvae. It is well known that biochemical metabolism is dramatically changed during the development of larval midgut in the 5th instar. In our current study, we are interested in ascertaining which proteins participate in midgut development in the penultimate instar larvae. Furthermore, when starvation is acted on animals, the animals will take violent responses to starvation by triggering emergent change in protein metabolism [12, 13]. These also can be revealed by proteomic analysis.

In the present study, we used 2-DE combined with MALDI-TOF-MS to analyze the midgut proteins of the 5th-instar larvae under normal and starvation conditions. As a result, 96 proteins were identified in this study and among them 69 proteins were observed for the first time. The results also indicated that the starved silkworm larval produced a 10 kDa heat shock protein and a diapause hormone precursor in response to this disadvantage condition.

## 2. Materials and Methods

### 2.1. Insect and Protein Preparation

The silkworm strain *p50* (DaZao) used in this study was provided by the Institute of Sericulture and System Biology at the Southwest University of China. The larvae were reared on the fresh mulberry leaves at 25°C to the fifth instar day 3. Then, the larvae were randomly divided into two groups. The larvae in group I still were reared on fresh mulberry leaves, while those in group II were starved until wandering stage (about 240 h). Larvae randomly sampled from each group were dissected at 24-hour intervals, and the midguts were washed with 0.75% NaCl solution and stored at −20°C. Larval midguts were ground to powder in liquid nitrogen with mortar and pestle. The proteins from silkworm midguts were extracted with 1 mL lysis buffer (8 M urea, 4% (m/v) 3[(3-cholamidopropyl) dimethylammonio]-1-propane sulphonate (CHAPS, Sangon, shanghai, China), and 4% (m/v) dithiothreitol (DDT, BBI, Canada)) at 4°C for 40 min. After centrifuging twice at 12,000 g for 15 min, the supernatants were collected for proteomics analysis. The concentration of proteins was measured by Bradford kit as described in manufacturer's instructions (TIANGEN, Beijing, China).

### 2.2. Two-Dimensional Gel Electrophoresis

The midgut proteins were separated by two-dimensional electrophoresis according to the manufacturer's instructions (GE Healthcare, USA). Briefly, proteins were dissolved in rehydration solution (8 M urea, 2% CHAPS, 0.28% DDT, and 1% IPG buffer pH 3–10). The protein solution was loaded onto an immobilized PH gradients strip (IPG strip, 18 cm, line, pH 3–10, GE Healthcare, Sweden). Isoelectric focusing (IEF) was performed as follows: 50 V for 12 h, 100 V for 1 h, 200 V for 1 h, 500 V for 30 min, 1000 V for 30 min, 3000 V for 30 min, and 8000 V for a total of 70000 Vhr (voltage multiplied by hours). The current did not exceed 50 mA per strip. After IEF, the strips were equilibrated in equilibration buffer I (50 mM Tris-HCl, pH 8.8, 6 mol/L urea, 30% glycerol, 2% SDS, and 1% DTT) for 15 min and then in buffer II (replaced 1% DTT with 2.5% iodacetamide) for another 15 min. The equilibrated gel strip was loaded onto a 12.5% SDS-polyacrylamide gel. Electrophoresis was carried out in an Ettan DALTsix vertical electrophoresis system (Amersham Biosciences, Sweden) at 10 mA per gel for approximately 1 h, and then switched to 40 mA per gel until the bromophenol blue reached the bottom of the gel. Protein was visualized by silver staining.

### 2.3. Gel Image Analysis

The resultant 2D gels were scanned by Image Scanner III (GE Healthcare, USA) with LabScan 6.0 software (GE Healthcare, USA) at an optical resolution of 300 dpi. The scanned gels were analyzed using the ImageMaster 2D platinum 6.0 (GE Healthcare, USA) software supplied by the manufacturer, and the parameters for spots detecting were set to smooth: 2–5, minarea: 30–50, and saliency: 5–15.

### 2.4. In-Gel Digestion and MALDI-TOF-MS Analysis

The protein spots excised from the gels were cut into small pieces (about 1 mm^3^) and transferred into 0.5 mL Eppendorf tubes. In-gel digestion was carried out according to what was previously described in [14]. In brief, 100 *μ*L of destaining solution (15 mmol/L K_3_[Fe(CN)_6_], 50 mmol/L Na_2_S_2_O_3_) was added to each tube for 10 min destaining. Then, the gel pieces were washed with ddH_2_O for 15 min for three times. The gel pieces were dehydrated with 100 *μ*L ACN until the gel pieces turned opaque white. The ACN was discarded after centrifugation. The gel pieces were dried at room temperature for more than 40 min. Fifty microgram trypsin (10 mg/mL in 25 mM NH_4_HCO_3_, PH 8.0, Sigma, USA) was added to each tube and the incubation was carried out at 37°C for 16–20 h. The tryptic peptides were extracted twice with 30 *μ*L 50% ACN/5% trifluoroacetic acid (TFA, Sigma, USA). The supernatants were pooled and dried in a Centrivap Cold Trap (LABCONCO, USA). Prior to mass spectrum analysis, the peptides were redissolved in 3 *μ*L of 50% ACN/0.5% TFA and equally mixed with saturated *α*-cyano-4-hydroxycinamic acid (CHCA, Sigma, USA). The mass spectra of the peptides were recorded on a Voyager DE PRO MALDI-TOF-MS (Applied Biosystems, USA). The database used in this analysis contained two parts of protein sequence: 6642 silkworm protein sequences downloaded from NCBI (http://www.ncbi.nlm.nih.gov/) with keyword of *Bombyx* and 14623 sequences generated by the annotation of silkworm genomic sequences (http://silkworm.swu.edu.cn/silkdb/doc/download.html). The analysis of mass spectra and the process of protein identification were carried out according to a previous study [14].

### 2.5. RNA Extraction and Real-Time PCR

Total RNA was extracted from the midgut tissues in two groups using Trizol reagent (Invitrogen, USA). The RNA samples were dissolved in RNase-free water, and the concentrations were measured using spectrophotometer (Amersham Biosciences, Sweden). According to the protocol, 3 *μ*g of total RNAs were used to synthesize the first-strand cDNA in 25 *μ*L of reverse transcription PCR system with AMV Reverse Transcriptase (Promega, USA). For real-time PCR, the primers designed for 10 kDa small heat shock protein gene were 5′-GTTCCTCTTCTGGACCGTG-3′ (forward) and 5′-GACCGACCGCTACTACTTCT-3′ (reverse), and for diapause hormone precursor gene were 5′-GGAAATGTACCAACCTGACCC-3′ (forward) and 5′-AACGCTTCTGGCAACCCTAT-3′ (reverse). Real-time PCR was performed in triplicate for each gene of interest in 20 *μ*L reactions with reverse transcription product, 2 × PCR buffer, 50 × ROX Reference Dye, 100 nM of each of the forward and reverse primer, and RNase free ddH2O. The PCR reaction was performed on an ABI Prism 7000 Sequence Detection System (Applied Biosystems) using the following program: initial denaturation at 95°C for 10 sec and 40 cycles of 95°C for 5 sec 60°C for 31 sec. *β*-actin3 was used as inner control.

## 3. Results

### 3.1.  2-DE Profiles of Silkworm Larval Midgut Proteins

**Figure 1:**
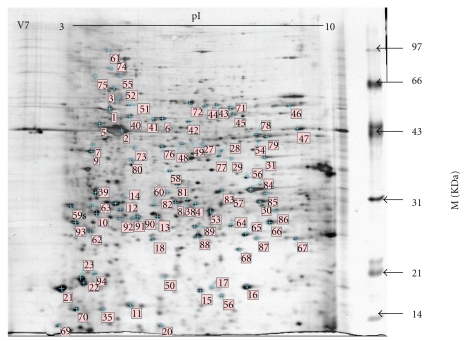
The 2-DE profile of midgut proteins at the fifth-instar day 7 larvae. A total of 300 *μ*g protein from midgut was applied onto a pH 3–10 Immobiline dryStrip (18 cm) for the first-dimensional electrophoresis and then separated by 12.5% SDS-PAGE. The 2-DE gels were stained by silver. Proteins identified in this study are indicated, and the numbers corresponded to those are in Table 1.

**Figure 2:**
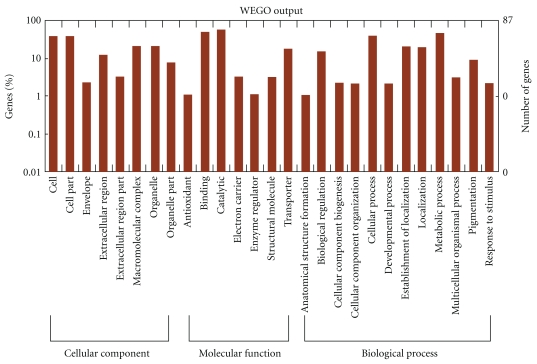
GO categories of the identified proteins by Wego software.

The silkworm midgut proteins were extracted from the fifth-instar day-4 (V4) to day-8 (V8) feeding larvae and from the fifth-instar day-4 (V4N) to day-13 (V13N) nonfeeding larvae. A total of 15 extractions were separated by 2D technology, as shown in Supplemental Data 1 and 2 in Supplemental Material available at doi:10.1155/2011/876064. The protein spots were then detected using ImageMaster 2D platinum 6.0 software. On the gels V4 to V8, 439, 461, 498, 606, and 620 protein spots were detected, respectively. And on the gels V4N to V13N, 420, 455, 500, 610, 630, 635, 643, 640, 660, and 684 protein spots were detected, respectively. The number of protein spots increased gradually with the development of larvae in two groups. As shown in Figure 1, the protein spots on the V7 gel were marked by number and further analyzed by a Voyager DE PRO MALDI-TOF-MS. After being searched by GPMAW software, proteins with more than five matched peptides and the coverage of peptides larger than 20% were accepted. The results were summarized in Table 1. A total of 96 proteins were identified. Among them, protein spots no. 95 and 96 were only found in nonfeeding midgut. When compared to the data obtained from the previous studies [10, 11, 15, 16], 69 proteins, accounting for 71% of all proteins, were found at the first time in our present study. Based on the molecular function and GO annotation (see below), these 96 proteins were classified into 12 categories including: (1) eight are for nutrition storage, (2) three for cell growth, (3) sixteen for protein metabolism, (4) three for carbohydrate metabolism, (5) nine for lipid metabolism, (6) eighteen for energy metabolism, (7) three are immune-related proteins, (8) two are antioxidation proteins, (9) eleven are muscle-related proteins, (10) five are for hormone metabolism, (11) sixteen for other functions, and (12) two are unannotated proteins. The identified proteins have been submitted to http://silkworm.swu.edu.cn/cgi-bin/wego/index.pl for GO (gene ontology) annotation. As shown in Figure 2, 96 proteins were classified into three groups including cellular component, molecular function containing binding, catalytic, transporter, electron carrier, and antioxidant, and biological processes involved in metabolism, cellular process, and biological regulation.

### 3.2. Changes in Protein Pattern in the Development of the Midguts of Normal-Feeding Silkworm Larvae

**Figure 3:**
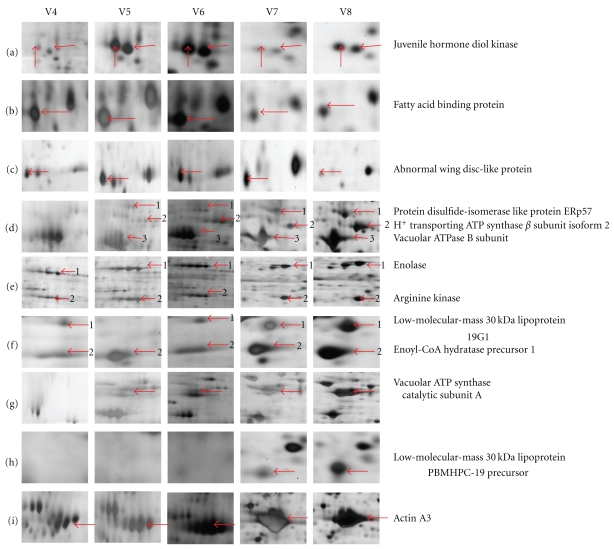
The partial enlargement of midgut proteins changed with development in normal feeding silkworm. Red arrows pointed out the target proteins and their names were displayed at right. Vx means fifth-instar day-x larvae.

As shown in Figure 1 and Supplemental data 1, the composition of the silkworm midgut proteins was different from V4 to V8. The number of protein spots, especially those ranging in size from 30 kDa to 90 kDa at higher pI region, gradually increased with the growth of larvae. Table 1 showed that the abundances of 64 midgut proteins were different from V4 to V8, mainly including those involved in nutrition storage, protein/lipid/energy metabolism, and muscle-related proteins. Most prominent among them were thirteen proteins with more than two-fold differential expression. Two spots corresponding to a juvenile hormone diol kinase (JHDK) and a fatty-acid-binding protein were present from V4 to V8, and displayed the highest expression level in V6 (Figures 3(a) and 3(b)). An abnormal wing disc-like protein had a peak in V7 (Figure 3(c)). From Figures 3(d) to 3(i), one can see that ten proteins among which two were low-molecular-mass 30 kDa lipoproteins 19G1 and PBMHPC-19 precursor, three were ATP synthase subunits, and five were, respectively, protein disulfide-isomerase-like protein (PDI) ERp57, enolase, arginine kinase, Enoyl-CoA hydratase precursor 1, and actin were expressed at relatively higher levels in V8. From Table 1, we also found that 6 proteins (spot no. 17, 19, 26, 33, 34, and 37), a 30 K lipoprotein precursor, an H+-transporting ATP synthase subunit, a cyclophilin, a glutathione S-transferase10, a NADPH oxidase, and an unknown protein, were barely detectable before metamorphosis in feeding larvae.

### 3.3. Midgut Proteins in Response to Starvation

After being fed with mulberry leaves for three days, a part of these fed silkworm larvae were separated for starvation treatment. The starved larvae survived for 10 days. Most of them were dead during starvation. At the day 13 (V13N), a few larvae struggled to pupate. The midguts were carefully collected at 24-hour intervals from the starved larvae until day 13 (V13N). We subsequently investigated these 10 protein samples from V4N to V13N by 2D electrophoresis coupled with MALDI-TOF MS. Supplemental Data 2 showed that the protein profiles of V4N and V5N were quite similar to each other, and the number of the protein spots on both gels was decreased as starvation time increased, compared to those in V4 and V5. However, the spot number was dramaticly increased at V6N compared to that in V5N. On the V6N to V12N gels, the numbers of protein spots were also increased and varied from 610 to 660. The protein distribution pattern of V6N to V12N resembled those of V5 to V8, suggesting that the starved larvae might keep their midgut intact and functional for a period of time. It is also important to note that the spot number was increased at V13N, up to 684, presumably coincident with the striking alteration of starved larvae at that stage.

**Figure 4:**
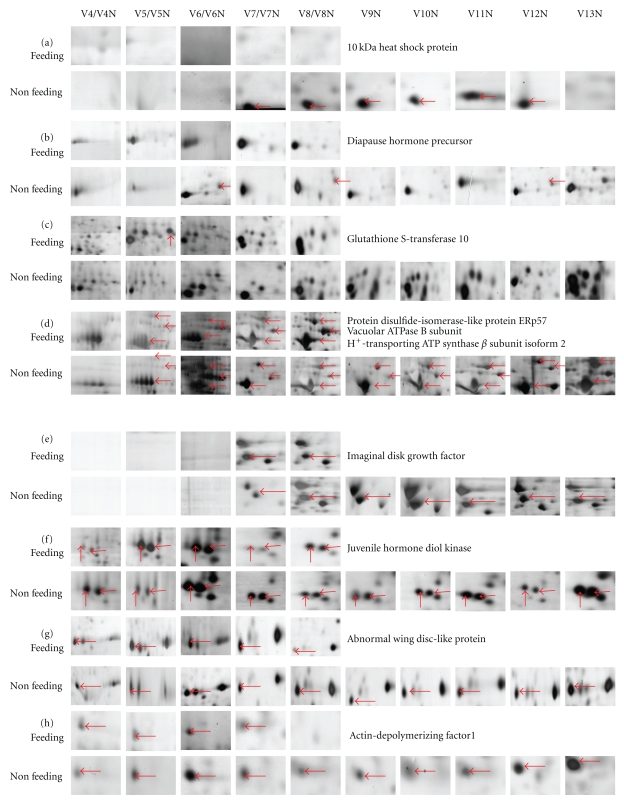
The partial enlargement of the midgut proteins in nonfeeding silkworms. Red arrows pointed out the target proteins and their names were displayed at right. Vx means fifth-instar day-x larvae.

The proteins identified in starved larval midgut were summarized in Table 1. As shown in Table 1, over 50% of identified proteins in nonfeeding silkworm larvae had a similar variation trend to that in normal feeding ones. On the contrary, 17 proteins delay their presentation compared with those in feeding group. Moreover, nine proteins (spot no. 29, 32, 36, 39, 50, 62, 63, 79, and 89) still present in nonfeeding larvae were undetectable before metamorphosis in feeding larvae; as a representative data, the expression pattern of actin-depolymerizing factor 1 was shown in Figure 4(g).

In Figures 4(a) and 4(b), a 10 kDa heat shock protein and a diapause hormone precursor were only detected in the nonfeeding groups. Real-time PCR was carried out to reveal changing expression patterns of two genes encoding the 10 kDa heat shock protein and diapauses hormone precursor. The data showed that this small heat shock protein gene was expressed in the normal feeding and nonfeeding larvae, but its peak signal can be detected after spending nine days in starvation (Supplementary Data 3A). As shown in supplementary data 3B, diapauses hormone precursor gene was expressed only in starved larvae at V5N and V13N.

Besides these two proteins, the glutathione S-transferase 10 was identified at V5 but not found from V4N to V13N (Figure 4(c)). The expressions of a PDI-like protein ERp57 and a vacuolar ATPase B subunit were upregulated at V6N and thereafter downregulated and returned to the same level as feeding silkworm (Figure 4(d)). The amounts of H+-transporting ATP synthase beta subunit isoform 2 and imaginal disc growth factor were at first lower in nonfeeding larvae but finally increased to the same level as in feeding larvae (Figures 4(d) and 4(e)). In Figure 4(f), we found that the amount of an abnormal wing disc-like protein decreased with development progress in feeding larvae, but after starvation, it kept a stable level (Figure 4(g)). 

## 4. Discussion

The domesticated silkworm, *Bombyx mori*, is a herbivorous insect and a model organism for Lepidoptera. Chinese and Japanese scientists made great efforts to accomplish the sequencing project of the whole silkworm genome [17, 18], which offers *Bombyx* researchers an opportunity to identify peptides using proteomic method. Midgut, the largest digestive organ in the silkworm body, is important for the proteomic research [10, 11]. Besides cuticle, silkworm midgut is another large interface between intra and extra. It is an entry for pathogens, toxins, and pesticides. Hence, any knowledge of protein components of silkworm midgut would be essential for developing new lepidopteran pest control strategies.

In this study, we used two-dimensional electrophoresis combined with MALDI-TOF-MS to analyse the midgut proteins in the normal fifth-instar silkworm larvae as well as in the starved larvae. The numbers of proteins in two groups were gradually increased from day 3 to the last larval day, suggesting that more and more midgut proteins may participate in the growth and metamorphosis of this tissue. Of the 96 proteins identified in this proteomics analysis, 69 proteins had not been observed prior to this study [10, 11, 15, 16], and the majority of midgut proteins detected showed developmental differences. For example, JHDK is responsible for the degradation of JH. Li et al. found that the mRNA of JHDK was expressed throughout 4th- and 5th-instar larvae at a constant level and distributed in foregut and midgut [19]. While JHDK protein reaches its peak at day 6 of 5th-instar larvae, it seems likely that it might be responsible for the decreased titer of JH at that stage. The fatty-acid-binding protein is involved in fatty acid transport. According to the microarray data, the gene for fatty-acid-binding protein was expressed in the 5th-instar silkworm larvae with a higher level at days 4-5, and then decreased to a constant level. The highest abundance of this protein was observed at day 6 on our 2-DE profiles, suggesting the active metabolism of lipids at that time. The abnormal wing disc-like protein detected was dramatically decreased at the day 8. It has the nucleoside diphosphate kinase activity and is involved in epithelial integrity [20]. Our observation indicated that it was regulated by hormone. Our analysis also led us to find a transferrin. Transferrin is a multifunction protein working in iron binding, transportation, cell growth, differentiation, and protection cells by inhibiting apoptosis [21].

Different insects have different responses to starvation. In some insects like *Manduca sexta*, the subsequent development of their larvae would be delayed when starved [22], but *Onthophagus taurus* larvae respond to food deprivation by shortening the length of the instar, becoming premature, pupating, and eclosing early to be small adults [23]. *Bombyx mori* belongs to the former type. When the 5th-instar silkworm larvae are starved from day 3, the duration of larval stage would be prolonged. It was found that the threshold weight is needed before metamorphosis in *Psacothea hilaris* [24]. This may be a reason why most of starved silkworm larvae were unable to pupate. 

Our data showed that nine proteins undetectable in feeding larvae before metamorphosis were still detected in nonfeeding silkworm. Of these proteins, actin-depolymerizing factor 1 plays roles in regulating F-actin organization, in cell and organ expansion [25]. More detailed analysis will uncover its function in silkworm midgut. Proteomic analysis also allowed us to identify two proteins only existing in the midgut of the starved silkworm larvae. One is a small heat shock protein and the other is a diapause hormone precursor. Small heat shock proteins are known to act as molecular chaperones to refold polypeptides trapped in protein aggregates and to degrade the misfolded proteins [26]. It has been reported that small heat shock protein has additional functions such as the regulation of programmed cell death and the promotion or inhibition of apoptosis to maintain homeostasis [27–31]. This small heat shock protein was undetectable in normal feeding larvae but can be detected in starvation ones, suggesting that it may play some roles in starved larvae for survival. Diapause hormone precursor which is relative to the synthesis of diapause hormone is another protein identified in the starved larvae. Insects can undergo diapause under extreme conditions. Gene encoding diapause hormone precursor is expressed by the induction of starvation, suggesting that, when confronts starvation, larva would stop its development against starvation for survival.

Interestingly, the starved larvae produced more JHDK than the normal feeding ones. The more JHDK, the lower JH titer. At the end of penultimate instar of larva, metamorphosis is characterized by a sharp decrease in JH. However, most of the starved larvae did not undergo metamorphosis, indicating that low level of JH titer associated with high level of ecdysteroid titer in nutrition state is essential for initiating larval-to-pupal metamorphosis.

Larval midgut is a main place where digestion action usually occurs due to digestive enzymes. In this study, we found that, in starvation situation, the digestive enzymes in larvae midgut were not disappeared. These digested enzymes such as glycosidase, amylase, and trehalase were synthesized, secreted, and trapped into the glycocalyx, as first proposed by Santos et al. [32].

Since the chemical pesticides cause serious environmental problems, new strategies for pest control need to be developed. So far, there have been two major methods for pests control via insect midgut [33]. One is to use Cry toxins from *B. thuringiensis* as biocontrol agents to lyse midgut epithelial. Another is to use RNAi technology. Selected RNAi targets in the midgut include mRNAs coding for vacuolar ATPase, ribosomal protein S4, actin, and *α*-tubulin [33]. Our present study provided more targets derived from midgut for the pest management.

## Supplementary Material

We used 2-DE technology to separate the midgut proteins in silkworms under feeding and non-feeding conditions. The proteins identified in this paper are indicated by numbers using the Image Master 2D platinum 6.0 software.

## Figures and Tables

**Table 1:** List of proteins identified from midgut of *Bombyx mori*.

Protein classification	Spot no.	Protein description	Accession number	MW (kDa)/ theory PI	Coverage (%)	V4/V4N (F/N)	V5/V5N (F/N)	V6/VN (F/N)	V7/V7N (F/N)	V8/V8N (F/N)	V9N (N)	V10N (N)	V11N (N)	V12N (N)	V13N (N)
Nutrition storage	8	30 kDa protein [*Bombyx mori*]	ACO35750	29.83/5.90	23.8	+/+	+/+	+/+	+/+	+/+	+	+	+	+	+
	37	30 K lipoprotein precursor [*Bombyx mori*]	CAA30433	30.26/7.08	32.6	−/−	+/−	+/+	−/−	−/−	−	−	−	−	−
	38	Low-molecular-mass 30 kDa lipoprotein 21G1Precursor [*Bombyx mori*]	CAA38531	32.98/6.19	26.5	−/−	−/−	−/−	+/+	+/+	+	+	+	+	+
	57	Low-molecular-mass 30 kDa lipoprotein 19G1 [*Bombyx mori*]	CAA38533	32.41/9.58	65.5	−/−	−/−	−/−	+/−	+/+	−	−	−	−	−
	60	Low-molecular-mass 30 kDa lipoprotein 21G1 Precursor [*Bombyx mori*]	Q00801	30.35/6.38	50.2	−/−	−/−	−/−	+/+	+/+	+	+	+	+	+
	82	Low molecular 30 kDa lipoprotein PBMHPC-19 precursor [*Bombyx mori*]	NP_001095197	29.41/7.20	33.9	−/−	−/−	−/−	+/++	++/+	+	+	+	+	+
	85	Low-molecular-mass 30 kDa lipoprotein 19G1 [*Bombyx mori*]	CAA38533	29.56/7.91	39.1	+/−	−/+	+/+	+/+	+/+	+	+	+	+	+
	90	Mature 30 K lipoprotein [*Bombyx mori*]	CAA30434	28.57/6.37	44.0	−/−	−/−	−/−	+/+	+/+	+	+	+	−	+

Cell growth	45	Imaginal disk growth factor [*Bombyx mori*]	BAF73623	48.39/7.79	28.3	−/−	−/−	−/−	++/+	++/++	++	++	++	++	++
	50	Actin-depolymerizing factor1 [*Bombyx mori*]	NP_001093278	17.23/6.19	41.2	+/+	+/+	+/+	+/+	−/+	+	+	+	+	+
	75	Transferrin [*Bombyx mori*]	NP_001037014	77.20/6.84	27.5	−/−	+/+	+/+	+/+	+/+	+	+	+	+	+

Protein metabolism	3	60 kDa heat shock protein, mitochondrial [*Drosophila melanogaster*]	NP_511115	61.23/5.37	23.6	+/+	+/+	+/+	+/+	+/+	+	+	+	+	+
	7	Protein phosphatase 1 catalytic subunit [*Bombyx mori*]	NP_001040480	32.28/5.80	40.5	+/+	+/+	+/+	+/+	+/+	+	+	+	+	+
	18	Acireductone dioxygenase [*Aedes aegypti*]	XP_001649899.	21.91/4.85	40.8	+/−	+/−	+/+	+/+	+/+	+	+	+	+	+
	28	Glyoxylate reductase/hydroxypyruvate reductase [*Culex quinquefasciatus*]	XP_001865660	37.34/7.94	32.4	−/−	+/−	+/+	+/+	+/+	+	+	+	+	+
	40	Protein disulfide isomerase [*Aedes aegypti*]	ABF18378	46.34/5.11	33.4	−/−	−/−	+/+	+/+	+/+	+	+	+	+	+
	43, 44	DnaJ-like protein [*Bombyx mori*]	NP_001040185	59.96/7.32	29.9	−/−	−/−	−/−	+/+	+/+	+	+	+	+	+
	46	Dihydrolipoamide dehydrogenase [*Bombyx mori*]	AAM93255	52.94/8.90	27.8	−/−	−/−	−/−	+/+	+/+	+	+	+	+	+
	47	Aspartate aminotransferase [*Bombyx mori*]	ABF51214	40.32/5.87	32.3	−/−	−/−	−/−	+/−	+/+	+	+	+	+	+
	52	Protein disulfide-isomerase-like protein ERp57 [*Bombyx mori*]	NP_001036997	55.48/5.17	37.1	−/−	+/+	+/+++	++/+	++/+	+	+	+	+	++
	58	Ribosomal protein P0 [*Bombyx mori*]	NP_001037123	34.24/5.61	41.8	−/−	−/−	−/−	+/+	+/+	+	+	+	+	+
	63	Ubiquitin carboxyl-terminal hydrolase [*Drosophila melanogaster*]	NP_476940	25.52/4.87	29.6	+/+	+/+	+/+	+/+	−/+	+	+	+	+	+
	70	Ribosomal protein P2 [*Bombyx mori*]	NP_001037213	21.20/5.79	38.3	+/+	+/+	+/+	+/+	+/+	+	+	+	+	+
	74	Heat shock protein 70 [*Bombyx mori*]	NP_001036837	73.21/4.98	26.0	−/−	+/+	+/++	+/+	++/+	+	++	++	++	++
	87	Peptidylprolyl isomerase B [*Bombyx mori*]	NP_001040479	22.39/8.91	42.9	−/−	−/−	−/−	+/+	+/+	+	+	+	+	+
	95	10 kDa heat shock protein, mitochondrial [Lepeophtheirus salmonis]	ACO11796	11.18/7.68	49.5	−/−	−/−	−/−	−/+	−/+	+	+	+	+	−

Carbohydrate metabolism	6	Enolase [*Bombyx mori*]	NP_001091831	47.19/5.55	41.6	+/+	+/+	+/+	+/+	+/+	+	+	+	+	+
	13	Triosephosphate isomerase [*Bombyx mori*]	AAU34185	26.94/5.60	50.8	+/+	+/+	+/+	+/+	+/+	+	+	+	+	+
	31	Glyceraldehyde-3-phosphate dehydrogenase [*Bombyx mori*]	NP_001037386	35.42/8.55	29.2	−/−	+/−	+/+	+/+	+/+	+	+	+	+	+

Lipid metabolism	30	Enoyl-CoA hydratase precursor 1 [*Bombyx mori*]	ABD36107	32.13/8.33	45.9	−/−	+/−	+/+	+/+	+/+	+	+	+	+	+
	35	Fatty acid binding protein [*Bombyx mori*]	NP_001037574	15.01/5.08	24.2	+/+	+/+	++/++	+/++	+/+	+	++	++	+	++
	36	Apolipophorin-III precursor [*Bombyx mori*]	AAB02852	20.82/9.53	43.0	+/+	+/+	+/+	+/+	−/+	+	+	+	+	+
	48	Acyl-coenzyme A dehydrogenase [*Bombyx mori*]	NP_001037672	47.53/7.16	22.7	−/−	−/−	−/−	+/+	+/+	+	+	+	+	+
	54	Acyl-coenzyme A dehydrogenase [*Aedes aegypti*]	EAT42766	35.81/7.18	44.5	−/−	−/−	−/−	+/−	+/+	+	+	+	+	+
	56	3-hydroxy acyl-CoA dehydrogenase [*Bombyx mori*]	NP_001040414	33.80/8.87	28.4	−/−	−/−	−/−	+/+	+/+	+	+	+	−	+
	65	Lysophospholipase [*Bombyx mori*]	NP_001040255	25.97/6.94	36.2	−/−	−/−	−/−	+/+	+/+	+	+	+	+	+
	79	Acetoacetyl-CoA thiolase [*Bombyx mori*]	NP_001093296	40.00/7.56	42.6	−/−	−/−	−/−	+/+	−/+	+	+	+	+	+
	91	Phosphatidylethanolamine binding protein isoform 2 [*Bombyx mori*]	NP_001093267	21.92/5.94	47.7	−/−	−/−	−/−	+/+	+/+	+	+	+	−	+

Energy metabolism	1	H+-transporting ATP synthase beta subunit isoform 2 [*Bombyx mori*]	NP_001041705	54.89/5.20	37.8	+/+	+/+	+/+	+/+	+/+	+	+	+	+	+
	5	H+-transporting ATP synthase beta subunit isoform 1 [*Bombyx mori*]	NP_001040450	55.04/5.13	33.9	+/+	+/+	+/+	+/+	+/+	+	+	+	+	+
	15	Abnormal wing disc-like protein [*Bombyx mori*]	NP_001093284	17.37/7.30	48.1	++/++	++/++	+++/++	++/++	+/+++	++	+++	+++	+	+++
	20	Vacuolar ATP synthase subunit F [*Bombyx mori*]	NP_001040448	13.92/5.92	50.0	+/−	+/−	+/+	+/+	+/+	+	+	+	+	+
	22	Cytochrome c oxidase subunit Va [*Bombyx mori*]	NP_001106742	17.24/5.59	31.8	+/+	+/+	+/+	+/+	+/+	+	+	+	−	+
	23	H+-transporting ATP synthase delta subunit [*Bombyx mori*]	NP_001093091	17.16/5.66	49.1	+/+	+/+	+/+	+/+	+/+	+	+	+	+	+
	33	H+-transporting ATP synthase subunit d [*Bombyx mori*]	NP_001093279	20.20/5.49	39.7	+/+	+/+	+/+	−/−	−/−	−	−	−	−	−
	51	Vacuolar ATPase B subunit [*Bombyx mori*]	ACE78271	55.19/5.20	38.1	−/−	−/−	+/+++	+/+	++/+	+	+	+	+	++
	53	Ubiquinol-cytochrome c reductase [*Bombyx mori*]	ABF60225	29.43/8.52	23.1	−/−	−/−	−/−	+/+	+/+	+	+	+	+	+
	55	Vacuolar ATP synthase catalytic subunit A [*Bombyx mori*]	NP_001091829	68.60/5.13	38.7	−/−	+/+	+/++	++/+	++/++	++	++	++	++	++
	64	ATP synthase subunit alpha [*Bombyx mori*]	BAA03725	28.66/8.43	41.9	−/−	−/−	−/−	+/+	+/+	+	+	+	+	+
	66	ATP synthase subunit alpha [*Bombyx mori*]	BAA03725	28.66/8.43	41.9	−/−	−/−	−/−	++/+	++/+	+	+	+	++	++
	71	4-Hydroxybutyrate CoA-transferase, putative [*Aedes aegypti*]	EAT44921	43.62/6.61	28.1	−/−	−/−	−/−	+++/+	++/++	++	++	++	++	++
	76	Arginine kinase [*Bombyx mori*]	ACI01048	40.33/5.84	36.9	+/+	+/+	+/+	+/+	+/+	+	+	+	+	+
	77	Ubiquinol-cytochrome c reductase core protein II [*Bombyx mori*]	NP_001106225	46.18/9.55	22.4	−/−	−/−	−/−	+/+	+/+	+	+	+	+	+
	86	ATP synthase subunit alpha [*Bombyx mori*]	Q07405	28.66/8.43	41.9	−/−	−/−	−/−	+/+	+/+	−	−	−	+	+
	88	ATP synthase subunit alpha [*Bombyx mori*]	Q07405	28.66/8.43	41.9	+/+	+/+	+/+	+/+	+/+	+	+	+	+	+
	92	NADH dehydrogenase (ubiquinone) Fe-S protein 8 [*Bombyx mori*]	NP_001040316	25.49/6.14	31.2	−/−	−/−	−/−	+/+	+/+	+	+	+	−	+

Immune related	4	Immune-related Hdd13 [*Hyphantria cunea*]	AAD09281	28.38/6.86	60.8	+/+	+/+	+/+	+/+	+/+	+	+	+	+	+
	16	Cyclophilin A [*Bombyx mori*]	NP_001037301	18.10/7.97	73.9	+/+	+/+	+/+	+/+	+/+	+	+	+	+	+
	17	Cyclophilin [*Bombyx mori*]	ABA54173	18.12/7.97	62.4	+/+	+/+	+/+	+/−	−/−	−	−	−	−	−

Antioxidation	34	Glutathione S-transferase 10 [*Bombyx mori*]	NP_001108466	24.47/8.11	26.7	−/−	+/−	−/−	−/−	−/−	−	−	−	−	−
	89	Thiol peroxiredoxin [*Bombyx mori*]	AAR15420	22.08/6.10	35.9	+/+	+/+	+/+	+/+	−/+	+	+	+	+	+

Muscle related	2	Actin A3 [*Bombyx mori*]	CAA28192	42.23/5.38	31.9	+/+	+/+	+++/+++	+++/+	+++/++	++	++	++	++	+++
	9	Actin, muscle-type A1 [*Bombyx mori*]	Gi|113216|	42.27/5.19	29.3	+/+	+/+	+/+	+/+	+/+	+	+	+	+	+
	14	Actin, cytoplasmic A4 [*Bombyx mori*]	NP_001119727	30.05/5.80	24.7	+/+	+/+	+/+	+/+	+/+	+	+	+	+	+
	21	Myosin 1 light chain [*Lonomia oblique*]	AAV91411	22.77/4.31	33.0	+/+	+/+	+/+	+/+	+/+	+	+	+	+	+
	39	Actin, cytoplasmic A4 [*Bombyx mori*]	NP_001119727	30.05/5.80	25.5	−/−	−/−	−/−	+/−	−/+	+	+	+	+	+
	59	Beta-tubulin [*Bombyx mori*]	NP_001036887	25.18/4.51	30.8	+/+	+/+	+/+	+/+	+/+	+	+	+	+	+
	61	Myosin heavy chain [*Bombyx mori*]	ACQ72825	96.12/4.98	20.2	−/−	−/−	−/−	+/−	+/−	−	−	+	+	+
	62	Myosin light chain 2 [*Bombyx mori*]	ACF59735	22.04/4.52	32.8	−/−	−/−	−/−	+/−	−/+	+	+	+	+	+
	67	Muscular protein 20 [*Bombyx mori*]	NP_001040476	20.30/8.81	45.7	−/−	−/−	−/−	+/−	+/−	−	−	−	+	+
	68	Transgelin [*Bombyx mori*]	NP_001040372	20.91/8.40	85.0	−/−	−/−	−/+	+/+	+/+	+	+	+	+	+
	93	Myosin light chain 2 [*Bombyx mori*]	NP_001091813	22.04/4.52	30.3	−/−	−/−	−/−	+/+	+/+	+	+	+	+	+

Hormone metabolism	24, 25	Juvenile hormone diol kinase [*Bombyx mori*]	NP_001037080	20.79/4.42	39.5	+/+	+/+	+/+	+/+	+/+	+	+	+	+	+
	83	Putative farnesoic acid O-methyl transferase [*Bombyx mori*]	NP_001091815	26.83/6.73	35.1	−/−	−/−	−/−	+/+	+/+	+	+	+	+	+
	94	Putative farnesoic acid O-methyl transferase [*Bombyx mori*]	NP_001091815	24.26/5.03	28.7	+/+	+/+	+/+	+/+	+/+	+	+	+	+	+
	96	Diapause hormone precursor [*Bombyx mori*]	BAA03755	22.39/9.44	60.9	−/−	−/−	−/+	−/−	−/+	−	−	−	+	+

Other proteins	10	27 kDa glycoprotein, p27K [*Bombyx mori*]	Q8T113	25.58/4.95	25.1	+/+	+/+	+/+	+/+	+/+	+	+	+	+	+
	11	Cellular retinoic acid binding protein [*Bombyx mori*]	NP_001037364	14.97/5.53	56.8	+/+	+/+	+/+	+/+	+/+	+	+	+	+	+
	12	Stardust, isoform B [*Drosophila melanogaster*]	NP_572463	32.15/5.81	26.3	+/+	+/+	+/+	+/+	+/+	+	+	+	+	+
	19	NADPH oxidase [*Culex quinquefasciatus*]	XP_001859059	30.17/8.35	35.1	++/+	++/+	++/++	−/−	−/−	−	−	−	−	−
	29	Similar to CG31790-PA [*Tribolium castaneum*]	XP_971960	50.42/6.01	22.0	−/−	+/−	+/+	+/−	−/+	+	+	+	+	+
	32	Serpin-2 [*Bombyx mori*]	NP_001037021	41.86/4.72	21.1	−/−	+/+	+/+	+/+	−/+	+	+	+	+	+
	41	GE24263 [*Drosophila yakuba*]	EDW97530	47.04/5.95	29.3	−/−	−/−	−/−	+/+	+/+	+	+	+	+	+
	42	PREDICTED: hypothetical protein [*Nasonia vitripennis*].	XP_001602573	51.08/5.68	28.5	−/−	−/−	+/+	+/+	+/+	+	+	+	+	+
	49	AGAP011050-PA [*Anopheles gambiae*]	EAA45511	34.16/6.09	39.7	−/−	−/−	−/−	+/+	+/+	+	+	+	+	+
	69	GI18783 [*Drosophila mojavensis*]	EDW09939	18.98/4.74	39.9	+/+	+/+	+/+	+/+	+/+	+	+	+	+	+
	72	Mitochondrial aldehyde dehydrogenase [*Bombyx mori*]	NP_001040475	56.24/7.55	29.6	−/−	−/−	−/−	+/+	+/+	+	+	+	+	+
	73	Soluble guanylyl cyclase beta-1 subunit [*Manduca sexta*]	AAC61264	36.77/5.55	20.1	−/−	−/−	−/−	+/−	+/−	−	−	−	−	+
	78	Putative acetyl transferase [*Bombyx mori*]	BAH96561	42.01/7.84	31.0	−/−	−/−	−/−	+/+	+/+	+	+	+	+	+
	80	PREDICTED: similar to CG10122-PA [*Tribolium castaneum*]	XP_970918	43.23/5.03	20.2	−/−	−/−	+/+	+/+	+/+	−	−	+	−	+
	81	Ef1alpha-like factor isoform 1 [*Bombyx mori*]	NP_001040118	31.48/7.84	46.4	−/−	−/−	+/+	+/+	+/+	+	+	+	+	+
	84	Voltage-dependent anion-selective channel [*Drosophila melanogaster*]	NP_001033899	30.13/7.45	35.5	−/−	−/−	−/−	+/+	+/+	+	+	+	+	+

Unannotated	26	Unknown [*Bombyx mori*]	BGIBMGA004116	12.80/4.49	43.1	+/+	+/+	+/+	−/−	−/−	−	−	−	−	−
	27	Unknown [*Bombyx mori*]	BGIBMGA013360	53.34/5.80	33.1	−/−	+/−	+/+	+/+	+/+	+	+	+	+	+

Total	96					37/34	47/40	52/54	85/87	75/84	80	80	82	78	85

“+” and “−” indicate protein with and without expression, respectively. The number of “+” means the x-fold up expression compared with the “+”. “Vx” means fifth-instar day-x larvae, “F” means feeding, and “N” means nonfeeding.
