# Supplementary material for: Proteomic Analysis of Larval Midgut from the Silkworm (Bombyx mori)
Source: Comp Funct Genomics. 2011 May 18;2011:876064. doi: 10.1155/2011/876064 (PMC3115381; doi:10.1155/2011/876064)
Supplement: Supplementary file 1 — We used 2-DE technology to separate the midgut proteins in silkworms under feeding and non-feeding conditions. The proteins identified in this paper are indicated by numbers using the Image Master 2D platinum 6.0 software. [file 876064.f1.pdf]

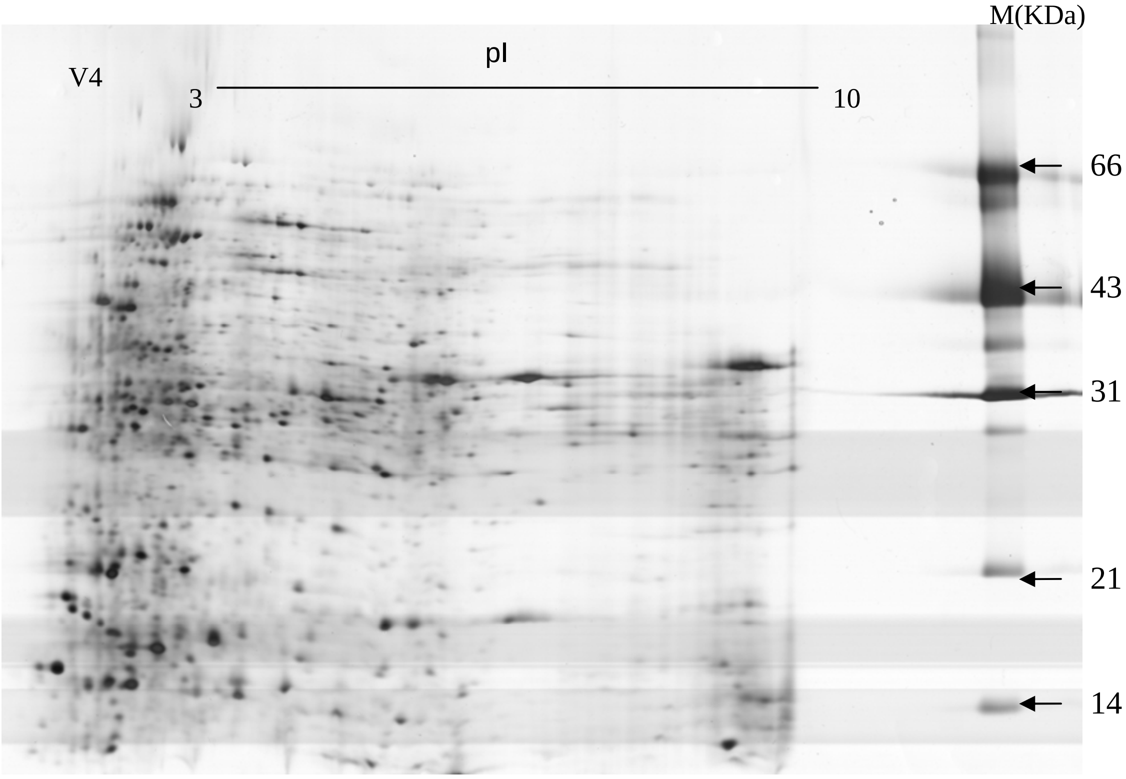
M(KDa)
V4
pI
3
10
66
43
31
21
14

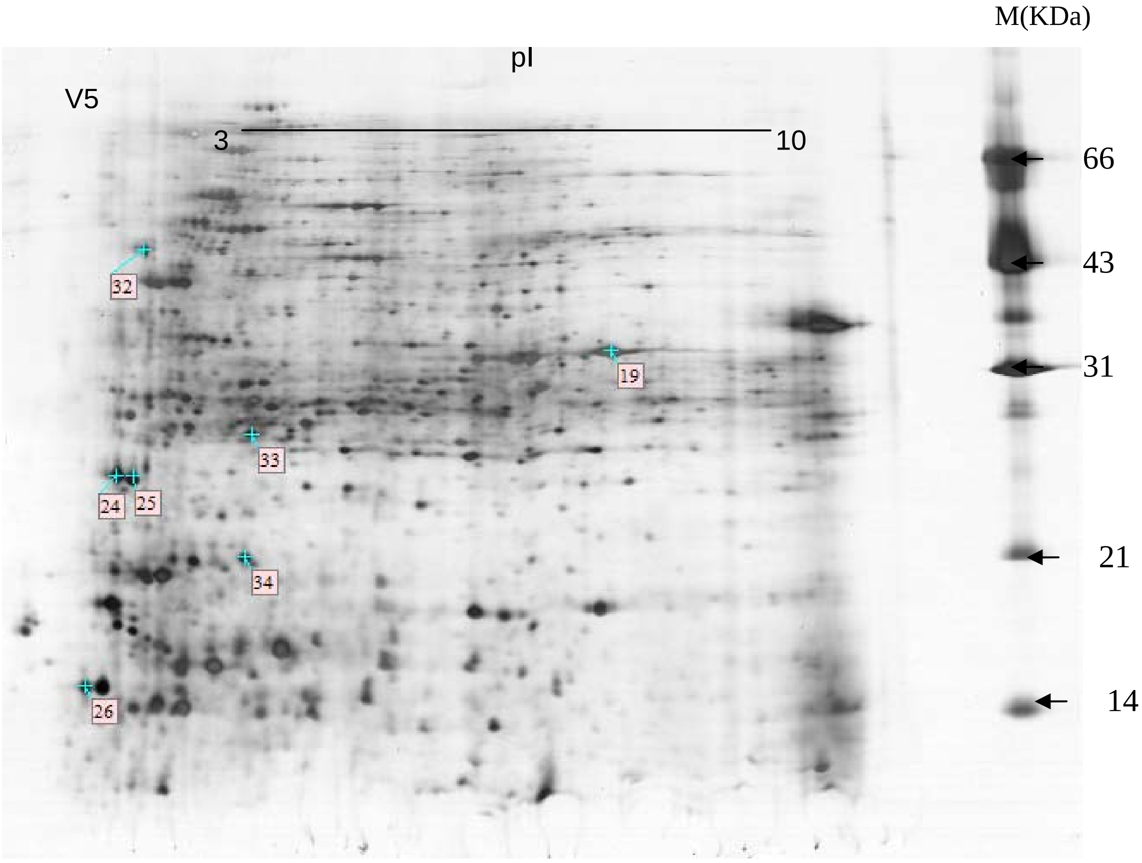
M(KDa)
V5
pI
3
10
66
43
31
21
14
32
19
33
24
25
34
26

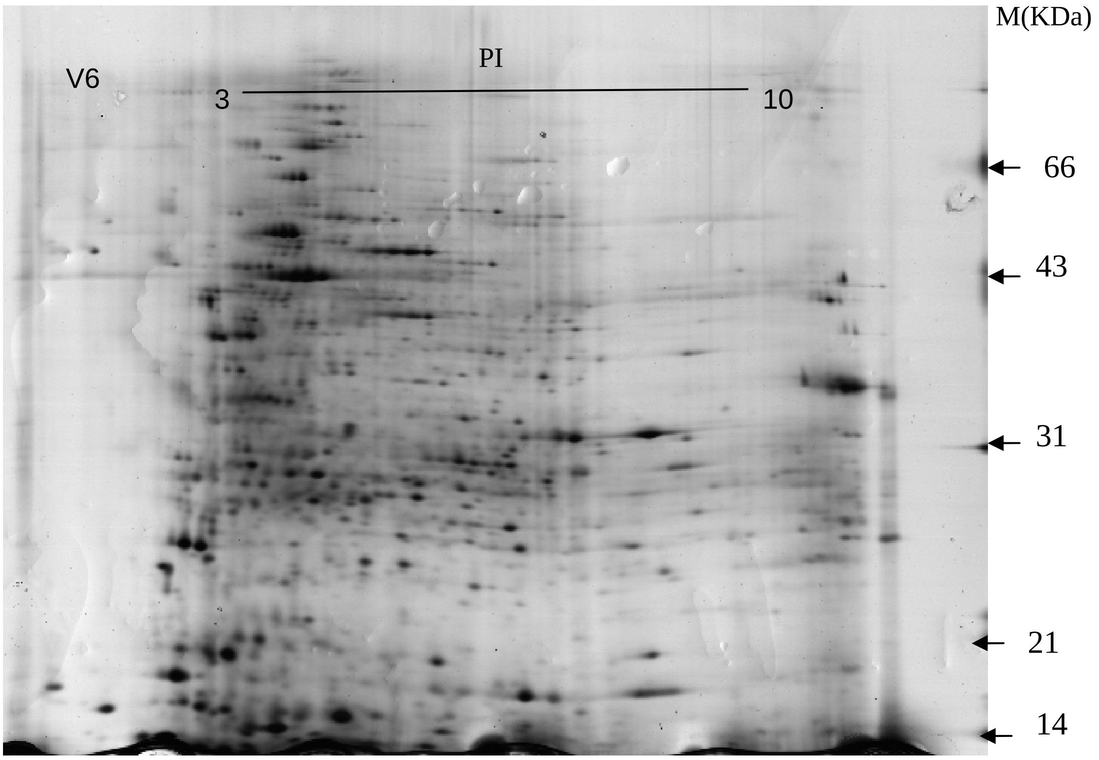
M(KDa)
PI
V6
3
10
66
43
31
21
14

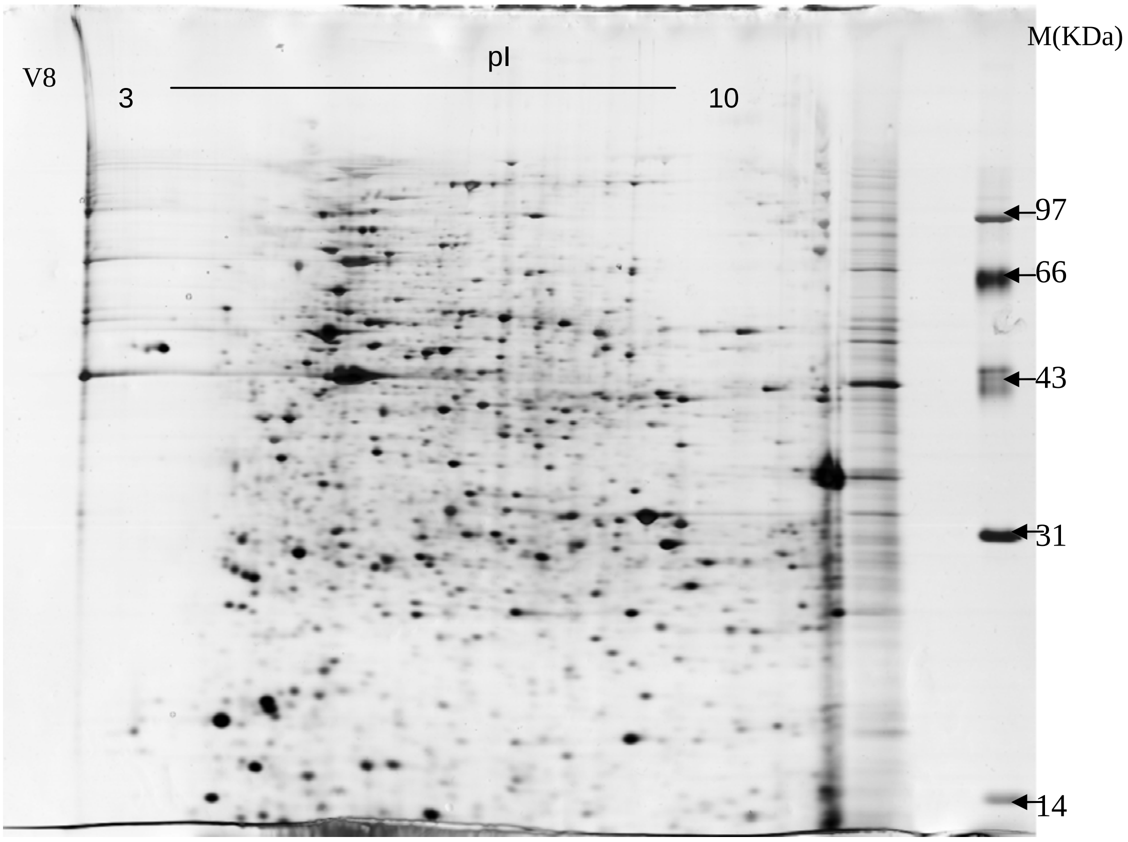
M(KDa)
pI
V8
3
10
97
66
43
31
14

Supplemental data 1 The 2-DE profile of midgut proteins at the fifth instar larvae day 4 to day 6 and day 8 in normal feeding silkworms. A total of 300 μg protein from each sample was applied onto a pH 3-10 immobiline drystrip (18cm) for the first-dimensional electrophoresis, and then separated by 12.5% SDS-PAGE. The 2-DE gels were silver-stained. Proteins identified in this study were indicated and the numbers corresponded to those in the table 1. VXF means fifth-instar day-x in normal feeding silkworm larvae.
