# Supplementary material for: Proteomic Analysis of Larval Midgut from the Silkworm (Bombyx mori)
Source: Comp Funct Genomics. 2011 May 18;2011:876064. doi: 10.1155/2011/876064 (PMC3115381; doi:10.1155/2011/876064)
Supplement: Supplementary file 2 [file 876064.f2.pdf]

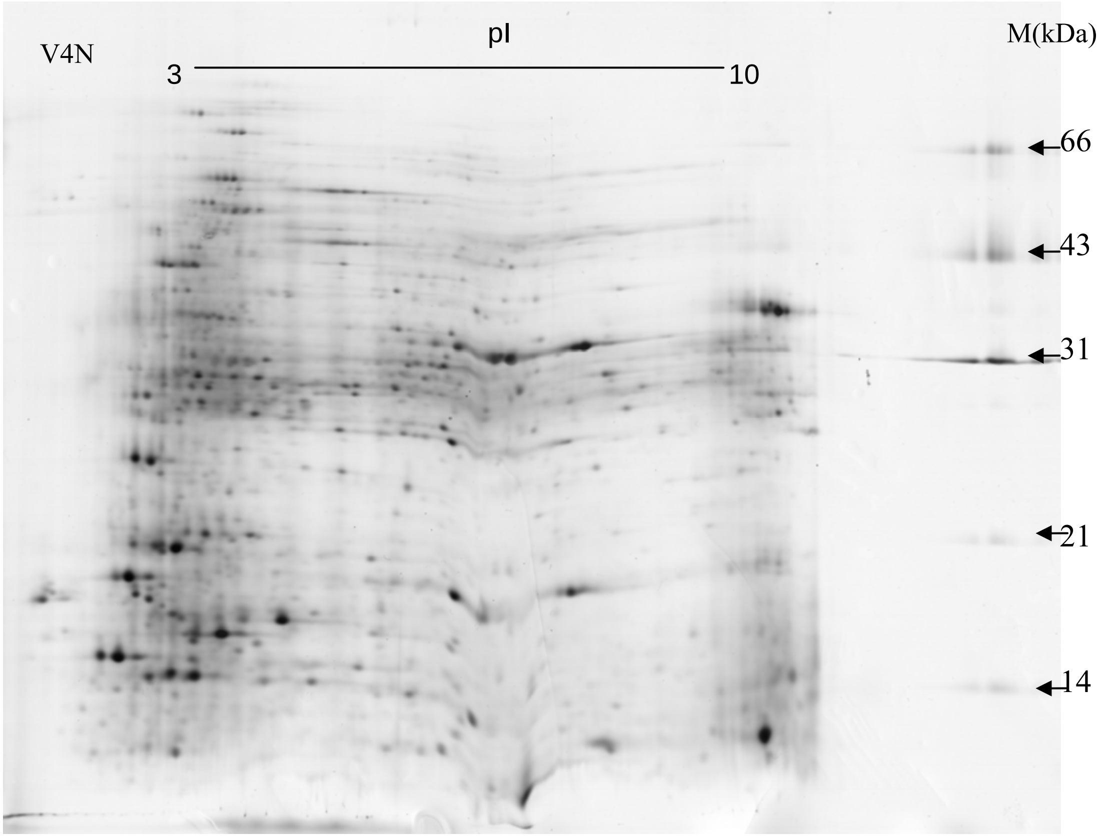
V4N
pI
3
10
M(kDa)
66
43
31
21
14

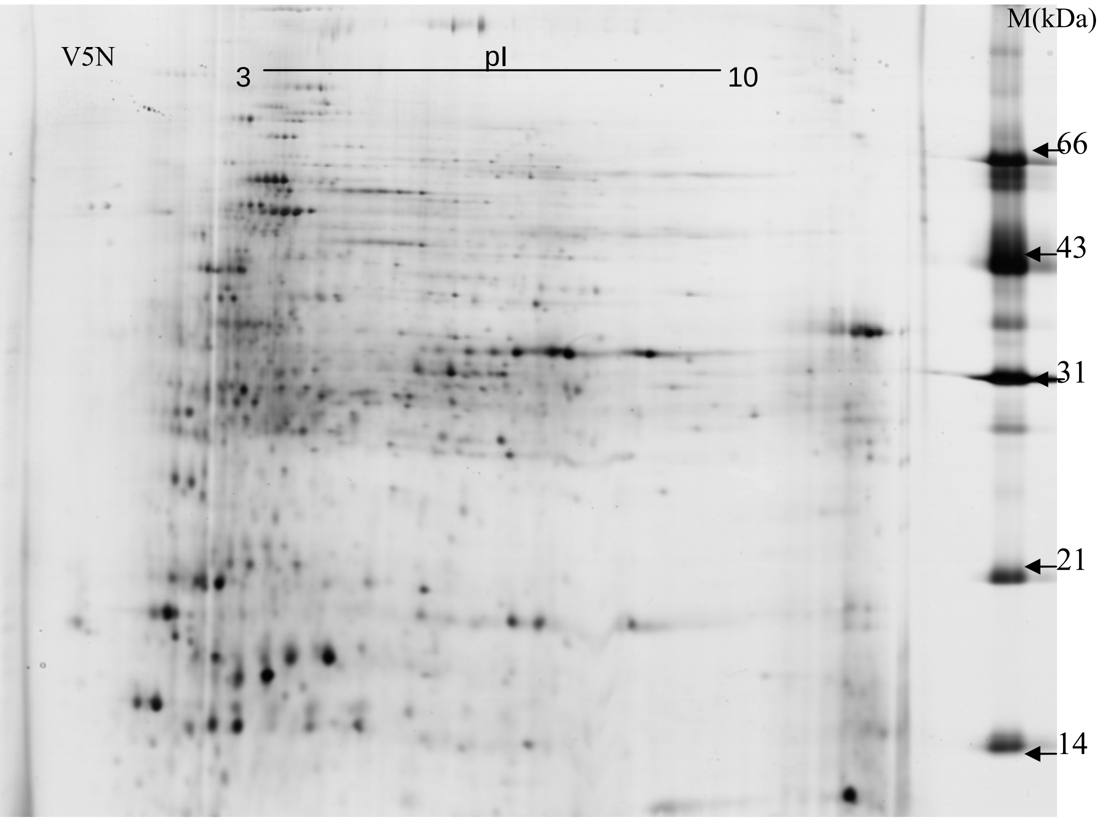
V5N
pI
3
10
M(kDa)
66
43
31
21
14

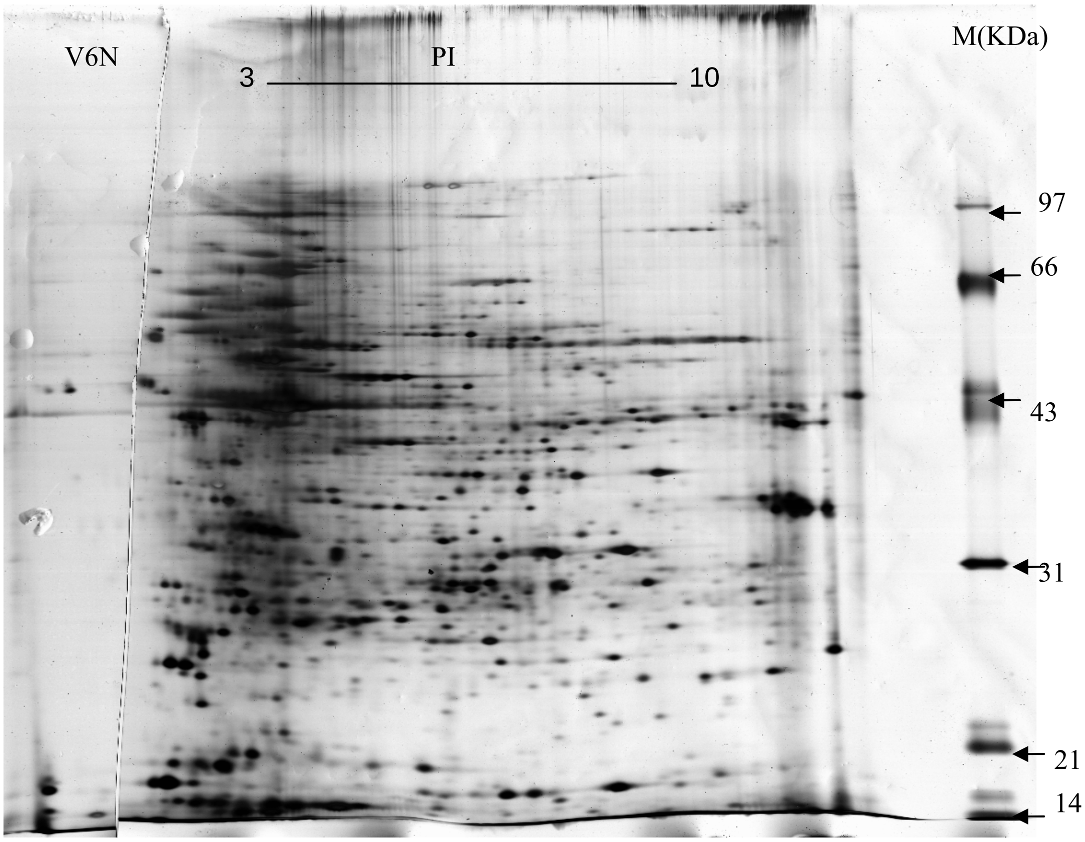
V6N
PI
3
10
M(KDa)
97
66
43
31
21
14

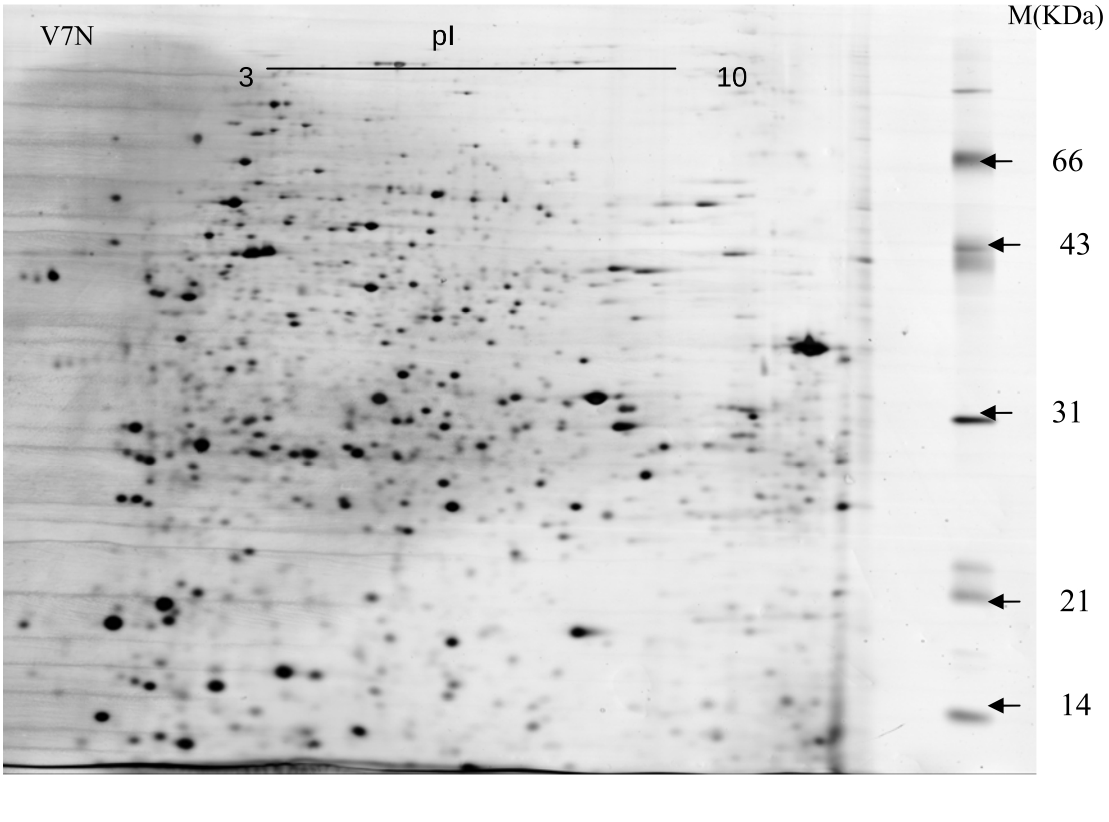
V7N
pI
3
10
M(KDa)
66
43
31
21
14

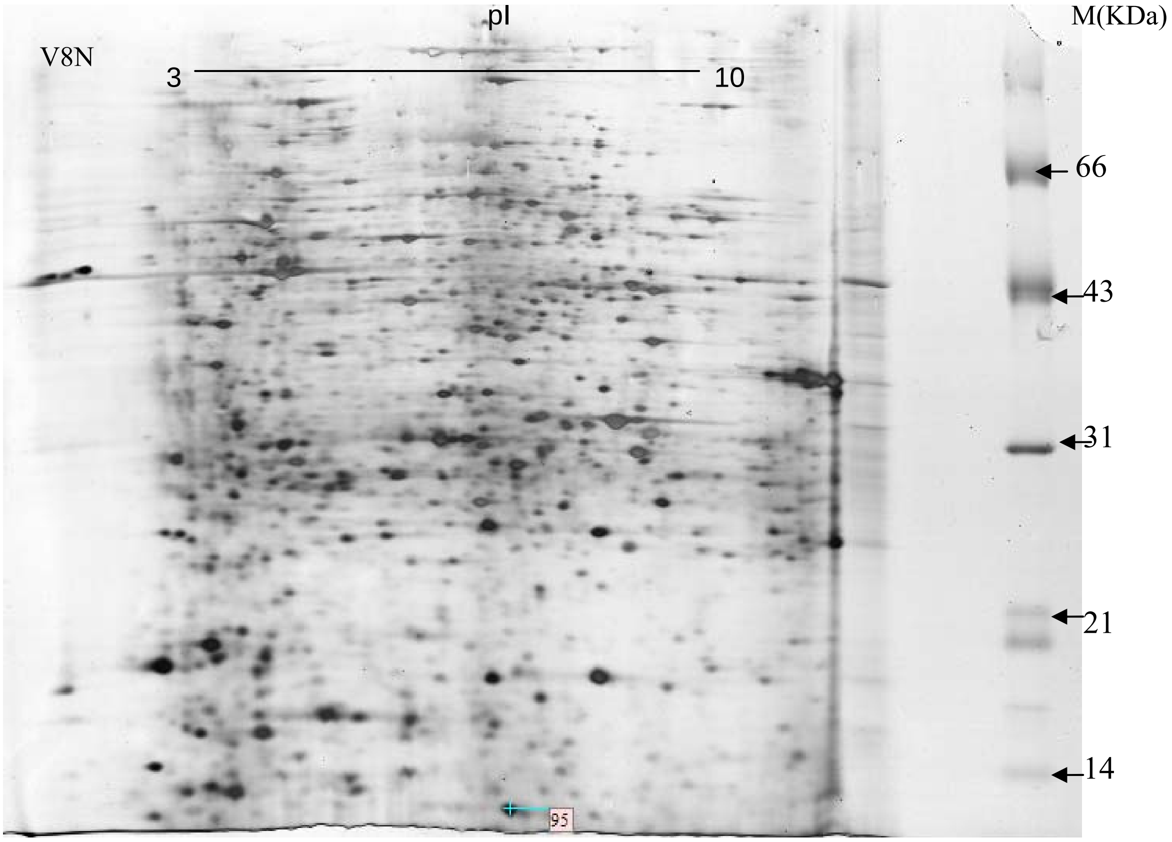
V8N
pI
3
10
M(KDa)
66
43
31
21
14

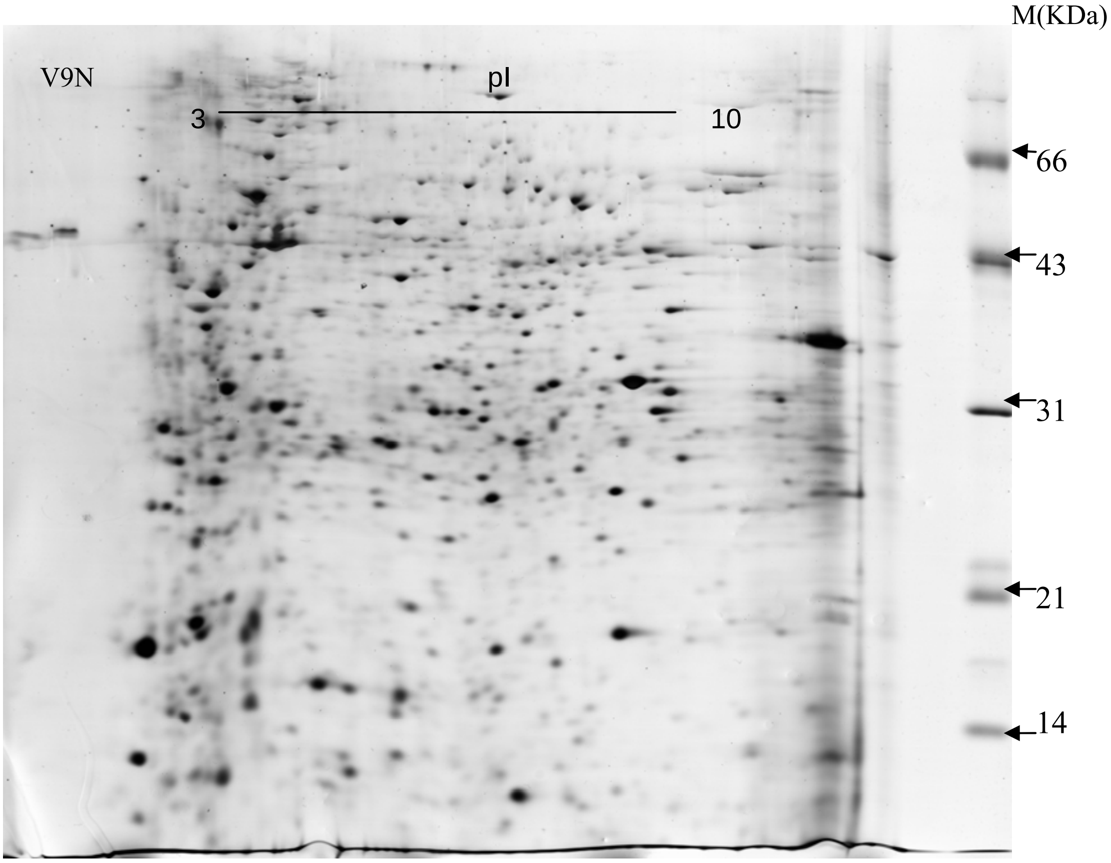
V9N
pI
3
10
M(KDa)
66
43
31
21
14

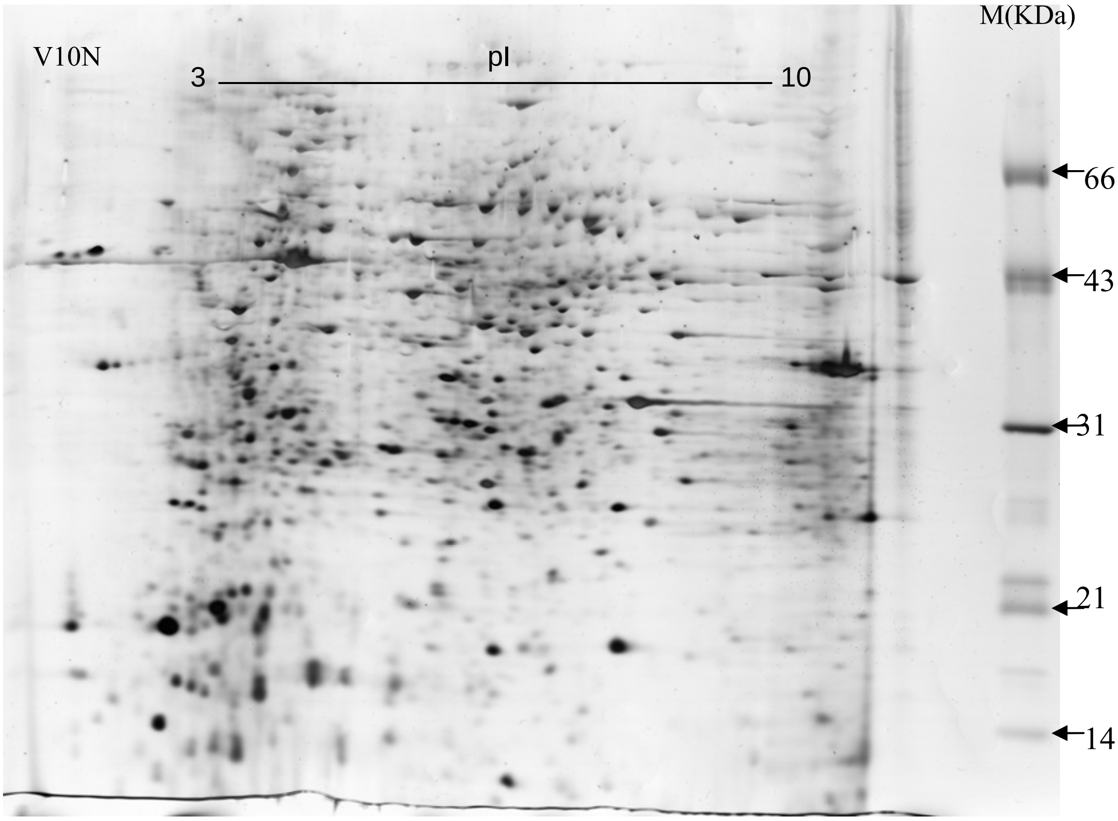
M(KDa)
V10N
pI
3
10
66
43
31
21
14

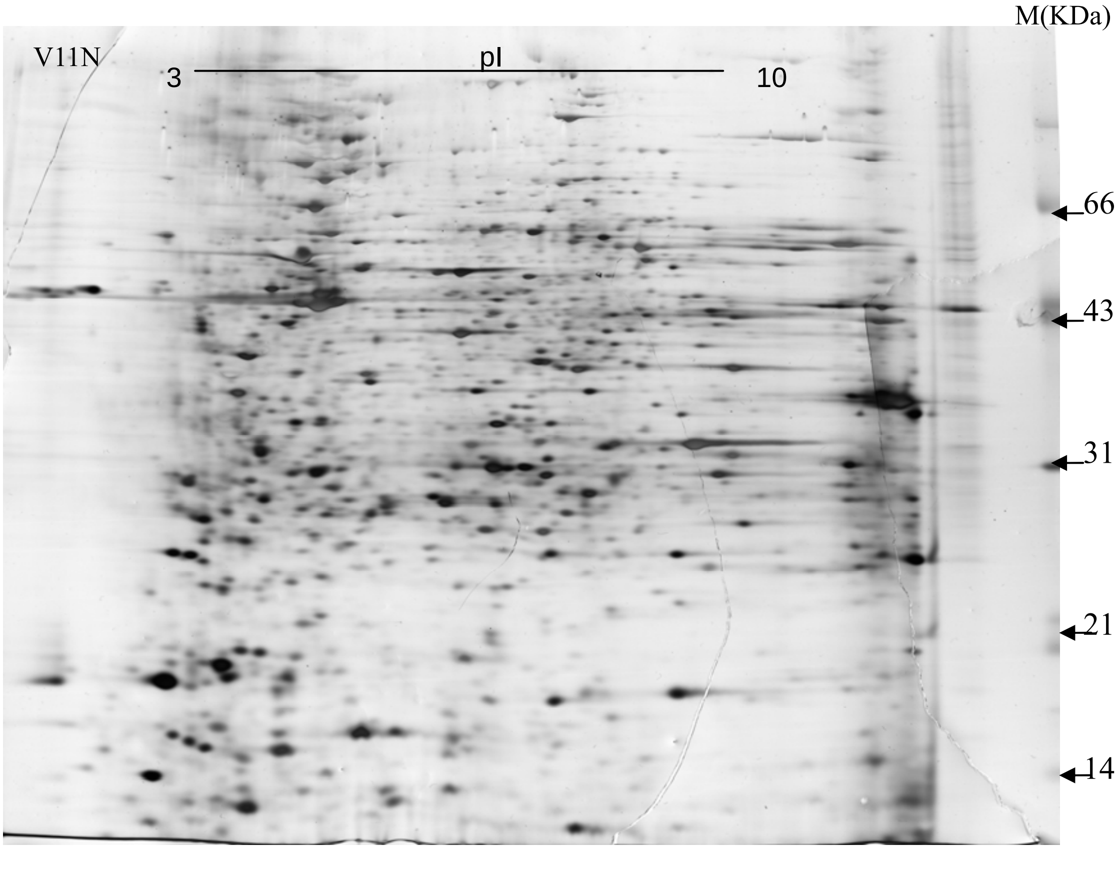
M(KDa)
V11N
pI
3
10
66
43
31
21
14

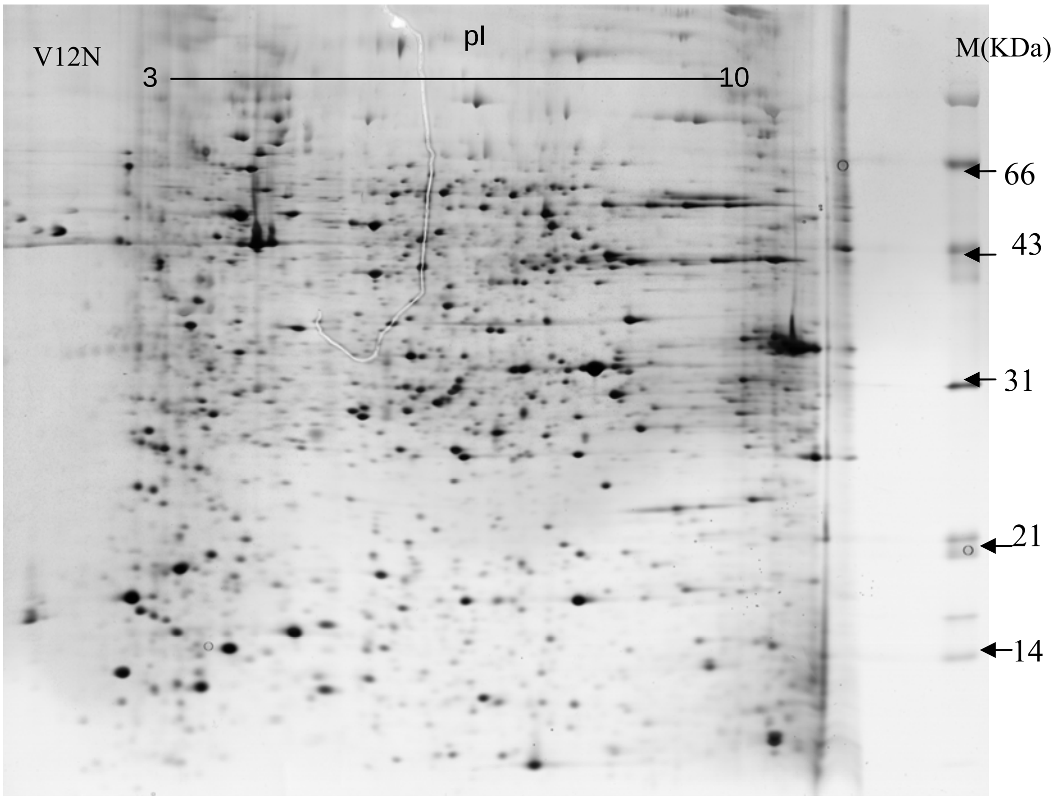
V12N
pI
3
10
M(KDa)
66
43
31
21
14

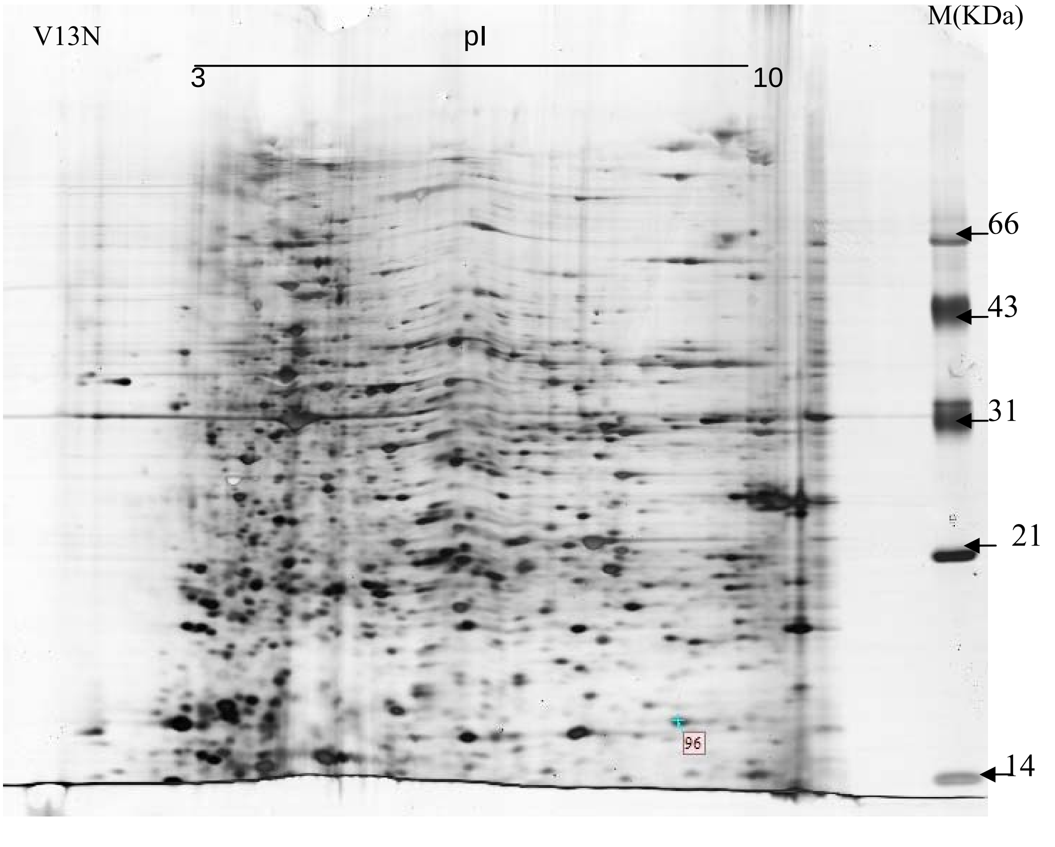
V13N
pI
3
10
M(KDa)
66
43
31
21
14
96

Supplemental data 2 The 2-DE profiles of midgut proteins at the fifth instar larvae day 4 to day 13 in non-feeding silkworms. A total of 300 μg protein from each sample was applied onto a pH 3-10 immobiline drystrip (18cm) for the first-dimensional electrophoresis, and then separated by 12.5% SDS-PAGE. The 2-DE gels were silver-stained. Proteins identified in this study were indicated and the numbers corresponded to those in the table 1. VXN means fifth-instar day-x in non feeding silkworm larvae.
