# Supplementary material for: Proteomic Analysis of Larval Midgut from the Silkworm (Bombyx mori)
Source: Comp Funct Genomics. 2011 May 18;2011:876064. doi: 10.1155/2011/876064 (PMC3115381; doi:10.1155/2011/876064)
Supplement: Supplementary file 3 [file 876064.f3.pdf]

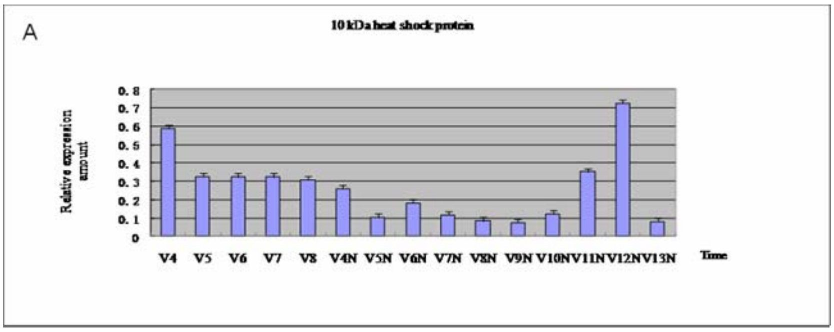

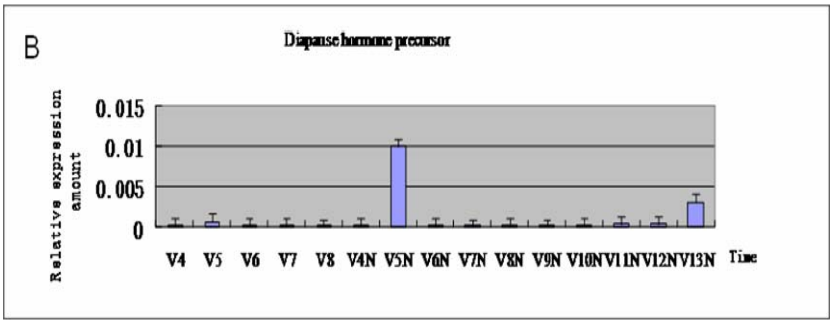

Supplemental data 3 Real-time PCR results of 10 kDa heat shock protein gene (A) and diapauses hormone precursor gene (B). The horizontal axis is different stages of normal feeding (V4-V8) and non-feeding larvae (V4N-V13N). Each bar represents the mean ±SD (n=3).
